# CHROMOMETHYLTRANSFERASE3/KRYPTONITE maintains the *sulfurea* paramutation in *Solanum lycopersicum*

**DOI:** 10.1073/pnas.2112240119

**Published:** 2022-03-24

**Authors:** Cláudia Martinho, Zhengming Wang, Andrea Ghigi, Sarah Buddle, Felix Barbour, Antonia Yarur, Quentin Gouil, Sebastian Müller, Maike Stam, Chang Liu, David C. Baulcombe

**Affiliations:** ^a^Department of Plant Sciences, University of Cambridge, Cambridge CB2 3EA, United Kingdom;; ^b^Epigenetics and Development Division, The Walter and Eliza Hall Institute of Medical Research, Parkville, VIC 3052, Australia;; ^c^Department of Medical Biology, The University of Melbourne, Parkville, VIC 3052, Australia;; ^d^Swammerdam Institute for Life Sciences, University of Amsterdam, Amsterdam 1098 XH, The Netherlands;; ^e^Institute of Biology, University of Hohenheim, 70599 Stuttgart, Germany

**Keywords:** paramutation, heredity, epigenetic memory, tomato

## Abstract

Paramutation involves the transfer of a repressive epigenetic mark from a silent allele to an active homolog and, consequently, non-Mendelian inheritance. In tomato, the *sulfurea* (*sulf*) paramutation is associated with a high level of CHG hypermethylation in a region overlapping with the transcription start site (TSS) of the *SlTAB2* gene that affects chlorophyll synthesis. The CCG subcontext hypermethylation is under-represented at this region relative to CTG or CAG, implicating the CHROMOMETHYLTRANSFERASE3a (CMT3) in paramutation at this locus. Consistent with this interpretation, loss of *CMT*3 function leads to loss of the *sulf* chlorosis, the associated CHG hypermethylation, and paramutation. Loss of *KRYPTONITE (KYP)* histone methyltransferase function has a similar effect linked to reduced H3K9me2 at the promoter region of *SlTAB2* and a shift in higher order chromatin structure at this locus. Mutation of the largest subunit of RNA polymerase V (*PolV*) in contrast does not affect *sulf* paramutation. These findings indicate the involvement of a CMT3/KYP–dependent feedback loop rather than the PolV-dependent pathway leading to RNA-directed DNA methylation (RdDM) in the maintenance of paramutation.

## Significance

Paramutation involves the transfer of a repressive epigenetic mark between silent and active alleles. It is best known from exceptional non-Mendelian inheritance of conspicuous phenotypes in maize but also in other plants and animals. Recent genomic studies, however, indicate that paramutation may be less exceptional. It may be a consequence of wide-cross hybridization and may contribute to quantitative trait variation or unstable phenotypes in crops. Using the *sulfurea* (*sulf*) locus in tomato, we demonstrate that a self-reinforcing feedback loop involving DNA- and histone-methyl transferases CHROMOMETHYLTRANSFERASE3 (CMT3) and KRYPTONITE (KYP) is required for paramutation of *sulf* and that there is a change in chromatin organization. These findings advance the understanding of non-Mendelian inheritance in plants.

Paramutation causes non-Mendelian inheritance in which an epigenetic mark at a silenced *paramutagenic* allele transfers to and silences an active *paramutable* homolog (1–3). In plants the DNA is methylated at the paramutated (silenced) sequence region, but this modification is not sufficient to mediate paramutation because many methylated loci do not show paramutation. Early examples of paramutation in maize (1), pea (4), and tomato (5) have striking phenotypes based on pigmentation or gross morphology. However, from genome-wide DNA methylation analyses, there is now evidence that paramutation-like effects may be more widespread (6–8). In some examples, the trigger for paramutation may be hybridization between distantly related varieties or even species (6–8).

Like all heritable epigenetic mutations, paramutation involves separate mechanisms to establish a molecular mark at the paramutable locus and to mediate its maintenance through cycles of cell division and sexual reproduction. Additionally, a defining characteristic of paramutation is the interaction between the participating alleles that, in many models, involves the RNA-directed DNA methylation (RdDM) pathway (1, 2).

RdDM is not, however, exclusive to paramutation. At many genomic loci, it mediates DNA methylation in transposable elements (TEs) and repetitive regions (9). It involves RNA polymerase IV (PolIV) transcription of the target DNA and RNA-dependent RNA polymerase (RDR2) conversion of the transcript into double-stranded RNA. DCL3 trims the double-stranded RNA into 24 nucleotide (nt) fragments and, after unwinding, the single-stranded 24-nt small RNAs (sRNAs) are loaded into Argonaute protein 4 (AGO4). This AGO4 nucleoprotein is then guided by Watson–Crick base pairing to an RNA polymerase V (PolV)–generated scaffold RNA, and it recruits DNA methyltransferases, including DRM2, that catalyze methylation of adjacent DNA cytosines (9).

The RdDM factors identified in paramutation screens in maize include the RDR2 ortholog (Mediator of Paramutation 1 [MOP1]), which is required to maintain and establish paramutation at multiple maize loci, including the classic paramutation example in the *booster1* (*b1*) locus (10). Maize genetic screens have also uncovered multiple PolIV subunits including MOP2/RMR7 (RDP2), MOP3, RMR6, RMR7, RDP1, and RDP2a as affecting paramutation (1). RPD2a has the potential to integrate PolV complexes (11). Consistent with involvement of the RdDM pathway, there may be abundant 24-nt sRNAs and high DNA methylation at the target loci. These 24-nt sRNAs could, in principle, diffuse within the nucleus and mediate the allelic interaction in paramutation.

However, the picture from genetic screens is incomplete. There is no clear evidence for involvement of the PolV subunits, AGO proteins, DNA methyltransferases, or other factors in the downstream part of the RdDM pathway or the 21-nt sRNAs or AGO6 associated with the initial establishment phase (12). Some maize studies also found it difficult to reconcile these findings with an sRNA-based model for paramutation (13). Paramutation at maize *purple plant1* (*pl1*), for example, can be established in mutants that lack sRNAs, including RMR1, an Snf2 protein that affects the stability of nascent transcripts (14). Conversely, mutations in the RMR12 chromodomain helicase DNA-binding 3 (CHD3) protein orthologous to *Arabidopsis* (*Arabidopsis thaliana*) PICKLE release the *pl1* maize paramutation but in a manner that probably promotes the incorporation of nucleosomes without affecting sRNA accumulation (15). These various findings and observations do not necessarily rule out the involvement of sRNAs or RdDM in paramutation, but they do point to the gaps in current models. Therefore, we are investigating *sulfurea* (*sulf)* paramutation in tomato (16). Our reasoning is that *sulf* paramutation may be mechanistically similar to the maize examples, but the genetic architecture of different plants would provide a new perspective on the factors involved.

The *sulf-*mediated leaf chlorosis phenotype is due to hypermethylation in a Differentially Methylated Region1 (DMR1) at the 5′ region of *SlTAB2 (TAB2),* a gene that affects the synthesis of chlorophyll in tomato plants (16). *TAB2* is silenced between 0 and 100% in the F1 progeny of *sulf* × wild-type (WT) plants (17). In crosses between variegated *sulf* lines and their parental WT lines, paramutation is poorly penetrant in F1 (<12%) (17). In F2, the percentage of variegation among heterozygous also varies, further showing that *sulf* has incomplete penetrance (5, 17). These differences in paramutation penetrance have been linked with the existence of different *sulf* epialleles with different degrees of paramutagenicity (5, 17).

Allele-specific DNA methylation analysis at DMR1 in interspecific *sulf X Solanum pimpinellifolium* F1 hybrids revealed high methylation levels in the CHG context in the de novo paramutated allele (16). In tomato, CHG-methylation is maintained by CHROMOMETHYLTRANSFERASE3a (*SlCMT3a*, hereafter referred as CMT3) (18). Here, we show that *sulf* paramutation requires the self-reinforcing loop involving CHG DNA methylation by the DNA methyl transferase CMT3 and H3K9 dimethylation by the histone methyltransferase KRYPTONITE (KYP) at the silenced locus, which correlates with changes in chromosome compartment. Mutation of the largest RNA polV subunit (NRPE1) does not affect *sulf.* Our findings suggest that the maintenance of *sulf* paramutation in tomato might be independent of the canonical RdDM pathway and that CHG methylation is required.

## Results

### CMT3 Maintains the *sulf* Paramutation.

**Fig. 1.**
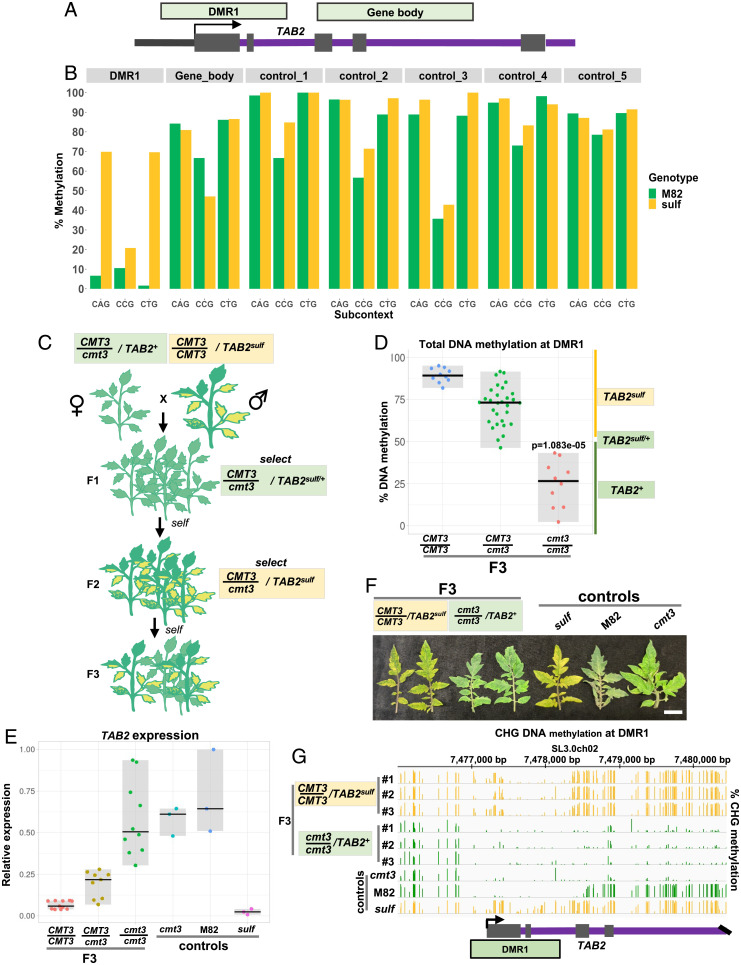
CMT3 maintains *sulf*. (*A*) Diagram illustrates *TAB2* locus and relative DMR1 and gene body positions. These regions were used for analysis in Fig. 1*B* (refer to *SI Appendix*, Table S1 for precise coordinates). (*B*) Mean % DNA methylation in leaf tissue at CHG subcontext in DMR1, *TAB2* gene body, and five random control regions within SL3.0ch02. M82: *S. lycopersicum* cv. M82 *TAB2^+^* (green), *n* = 2; and *sulf*: *S. lycopersicum cv. Lukullus TAB2^sulf^* (yellow), *n* = 2. Coordinates are listed in *SI Appendix*, Table S1. (*C*) Diagram illustrates crossing scheme used to generate F3 populations (F3 pedigree). (*D*) Jittered dots depict % of DNA methylation at DMR1 in individual plants determined by McrBC-qPCR. Plants denote F3 siblings–4-wk-old leaf tissue. The summary of the data is shown as the horizontal line indicating the median. Gray boxes illustrate the data range. The × axis refers to *CMT3* genotypes. *CMT3/CMT3 TAB2^sulf^, n* = 10; *CMT3/cmt3 TAB2^sulf^, n* = 30; *cmt3/cmt3 TAB2^+^, n* = 10. *P* value *cmt3/cmt3* versus *CMT3/CMT3* was calculated employing a Mann-Whitney–Wilcoxon test. (*E*) Jittered dots depict relative *TAB2* expression in individual plants (4-wk-old leaf tissue) normalized to the geometric mean of the expression of two reference genes (*SI Appendix*, Table S3). The summary of the data is shown as horizontal line indicating the median. Gray boxes illustrate the data range. F3 plants: *CMT3/CMT3 TAB2^sulf^, n* = 11; *CMT3/cmt3 TAB2^sulf^, n* = 8; *cmt3/cmt3 TAB2^+^, n* = 9. Controls: M82- (*S. lycopersicum cv. M82) CMT3/CMT3 TAB2^+^, n* = 3*; sulf-* (*S. lycopersicum cv. Lukullus) CMT3/CMT3 TAB2^sulf^, n* = 3*; cmt3- cmt3/cmt3 TAB2^+^, n* = 3. (*F*) Young leaves from 6-mo-old plants. White bar, 2 cm.1E. (*G*) *TAB2* Integrative Genomics Viewer (IGV) screenshot of bisulfite sequencing data exhibiting CHG DNA methylation (4-wk-old leaf tissue), range [0 to 100]; F3 plants and controls: same as in Fig. 1*E*. Green tracks refer to green leaf phenotype, and yellow tracks refer to plants that display chlorosis. (*A* and *C*–*G*) Yellow boxes refer to plants displaying *sulf* chlorosis, and green boxes refer to green plants.

The *sulf* phenotype results from *TAB2* hypermethylation in a DMR1 close to the transcription start site (TSS) (16). The severity of leaf chlorosis is directly proportional to the levels of DMR1 DNA methylation and thus far has not been associated with variation in DNA sequences (16). Here, we classify *sulf* epialleles based on their correlated phenotype and epigenotype (Table 1) (16). Despite displaying high differential DNA methylation at the CG context (∼50%), DMR1 is unusual in that it has a very high level of CHG hypermethylation (∼60%) (16) consistent with the involvement of CMT3 (18). To test this hypothesis, we assayed methylation at the different CHG subcontexts in DMR1 (Fig. 1*A*) (16) in leaf tissue excised from plants bearing *TAB2^+^* (WT cv. M82) or *TAB2^sulf^* (*sulf*). CMT3 preferentially methylates CAG and CTG motifs over CCG in multiple plant species, including tomato (18, 19). In *TAB2^sulf^*, the percentage of CCG methylation in DMR1 was lower than at CAG and CTG (Fig. 1*B*), consistent with the involvement of *CMT3* in *TAB2^sulf^*. The subcontext bias was less pronounced in the gene body of *TAB2* than in DMR1 (Fig. 1*B*). It was also less pronounced in random non-DMR regions of similar size within the pericentric heterochromatin of chromosome 2 (Fig. 1*B* and *SI Appendix*, Table S1).

**Table 1.** Epiallele classification employed in this study

Epigenotype	Genotype	DNA Methylation/Phenotype	Paramutated	Identification
***TAB2^+^***	M82 DNA sequence	<50% methylation, green	No	Phenotyping
				McrBC-qPCR
***TAB2^sulf/+^***	M82 DNA sequence	50% methylation, green (e.g., F1s)	No	Phenotyping
				McrBC-qPCR
***TAB2^sulf^***	M82 DNA sequence	>50% methylation, variegated leaf chlorosis	Yes	Phenotyping
				McrBC-qPCR

To further test this possibility, we took advantage of the plants bearing CRISPR-mediated deletion in the *SlCMT3*a gene—*cmt3*a (referred to as *cmt3*) (18). The heterozygous *CMT3*/*cmt3* was crossed to *CMT3*/*CMT3* plants with the *TAB2^sulf^* epiallele (Fig. 1*C*). In the F1 generation, we selected plants heterozygous for *cmt3* and, from McrBC-qPCR (16), for the *TAB2^sulf/+^* epialleles characteristic of the epiheterozygous state at DMR1 (Fig. 1*C* and *SI Appendix*, Fig. S1*A*). The epiheterozygous state is characteristic of a *sulf* allele with low paramutation penetrance. Some *sulf* epialleles have strong F1 penetration, whereas others, such as the one used here, only display paramutation symptoms in F2 (17). Because pure *sulf* homozygotes are lethal, it is likely that F2 chlorotic *TAB2^sulf^* plants underwent paramutation (5) during early growth of *TAB2^sulf/+^*.

In the F2 generation, only *CMT3* homozygotes or heterozygotes displayed the *TAB2^sulf^* phenotype and epigenotype (>50% methylation by McrBC-qPCR) (*SI Appendix*, Fig. S1*B*). The outcome was the same in reciprocal crosses in which the paternal plants carried *cmt3* and the maternal *CMT3*/*CMT3* plants carried the *TAB2^sulf^* epiallele (*SI Appendix*, Fig. S1 *C* and *D*). From this result, we conclude that DMR1 hypermethylation and CMT3 are required for establishment or maintenance of *sulf* paramutation. There was a similar genetic dependence on *CMT3* for the *TAB2^sulf^* epiallele in the F3 progeny of *CMT3*/*cmt3* F2 plants *TAB2^sulf^* (Fig. 1*D*). As in the F2, the homozygous *cmt3* F3 plants were green and had lower DMR1 DNA methylation levels than their *CMT3* siblings (Fig. 1*D*), indicative of the epiallele *TAB2^+^*. This observation suggests that CMT3 is required to maintain *sulf* paramutation that established in the F2.

Further support for a link between *sulf* chlorosis, DNA methylation, and CMT3 level comes from the analysis of DMR1 DNA methylation in the heterozygous *CMT3*/*cmt3* F2 and F3 plants. The distribution of DMR1 DNA methylation in these plants was skewed toward lower values than in *CMT3* homozygotes (*SI Appendix*, Fig. S1*B* and Fig. 1*D*), and in the F2, a smaller proportion of plants were *TAB2^sulf^* and chlorosis in *CMT3/cmt3* (∼17%) than in *CMT3*/*CMT3* (∼24%) (*SI Appendix*, Fig. S1*B*). Many of the green F2 *CMT3/cmt3* heterozygous plants may have inherited *TAB2^sulf^* and *TAB2^sulf/+^* but with incomplete maintenance of *TAB2^sulf^* and reversion to *TAB2^+^* due to reduced dosage of *CMT3*.

Consistent with the low DNA methylation levels of DMR1 in the F3 *cmt3* backgrounds, *TAB2* expression in these plants was higher than in the F3 *CMT3* homozygous and heterozygous siblings (Fig. 1*E*), although with more variation than in *CMT3* or *cmt3* lines with *TAB2^+^* that had not been crossed with *sulf* (Fig. 1*E*). The homozygous *cmt3* plants lacked *sulf* chlorosis, whereas their F3 *CMT3* siblings were chlorotic (Fig. 1*F* and *SI Appendix*, Fig. S2).

More detailed analysis of the F3 DNA methylation pattern by bisulfite sequencing analysis confirmed that, compared to M82, the CHG DNA methylation in *sulf* plants bearing the *TAB2^sulf^* allele was higher in DMR1 extending from 212 bp in the 5′ upstream region into intron 2 at 852 bp downstream of the TSS (16) (Fig. 1*G*). In the *cmt3 TAB2^+^* F3 progeny, the hyper CHG DMR1 was lost (Fig. 1*G*). There was also CHG hypomethylation of the transcribed DNA but no indication that this gene body DNA methylation influenced paramutation (Fig. 1*G*). From these patterns, we conclude that *sulf* is associated with CMT3-mediated CHG hypermethylation of DMR1.

In the CG context, the *sulf* hyper DMR was restricted to the DMR1 region, but the association with *sulf* was weaker than with CHG (*SI Appendix*, Fig. S3). The degree of hyper CG methylation was markedly lower in the *cmt3 TAB2^+^* F3 progeny than in the *CMT3 TAB2^sulf^* siblings (*SI Appendix*, Fig. S3). The CHH context did not exhibit significant hypermethylation in the TAB2 DNA of *sulf* plants (*SI Appendix*, Fig. S3). There was a region of CHH hypomethylation on the upstream side of DMR1 (*SI Appendix*, Fig. S3) corresponding to a Differentially Methylated Region2 (DMR2) (16), but it was not correlated with the *sulf* phenotype in the F3 progeny: The hypomethylation was lost to a varying extent irrespective of whether the plants were *CMT3* and *TAB2^sulf^* or *cmt3* and *TAB2^+^* (*SI Appendix*, Fig. S3). These results indicate that CHG methylation patterns, but not CG or CHH, primarily govern the *sulf* phenotype.

The *cmt3* mutants lose DMR1 hypermethylation and *sulf* chlorosis, but in principle, they could retain other epigenetic marks with the potential to reestablish *TAB2^sulf^* in a *CMT3* background. To assess this possibility, we backcrossed an F2 *cmt3*/*cmt3 TAB2^+^* plant with one of the highest levels of methylation (27%) (*SI Appendix*, Fig. S3*B*) with an M82 *TAB2^+^* allele (*SI Appendix*, Fig. S4*A*) and quantified DNA methylation at DMR1 using McrBC-qPCR in the BC1 and two generations of selfed progeny (*SI Appendix*, Fig. S4*A*). None of these plants had DMR1 DNA methylation levels higher than 40% (*SI Appendix*, Fig. S4 *B*–*D*), and plants were not chlorotic (*SI Appendix*, Fig. S4*E*). Bisulfite sequencing confirmed lower levels of CHG methylation at DMR1 in backcrossed plants than in *sulf* controls (*SI Appendix*, Fig. S5), suggesting that *sulf* memory is *CMT3* dependent and that RdDM is not sufficient to maintain *sulf.*

### NRPE1 and *sulf* Paramutation.

**Fig. 2.**
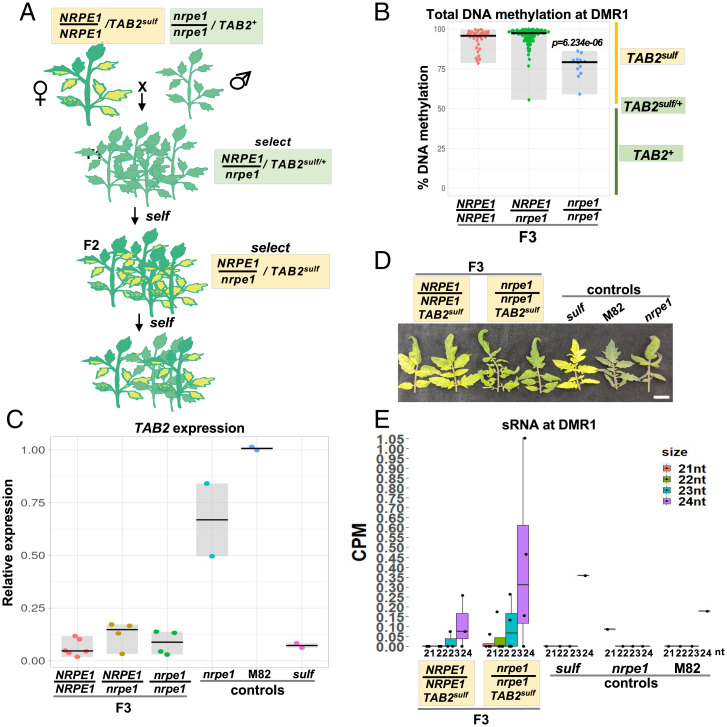
*sulf* maintenance remains unaffected in *nrpe1* mutants. (*A*) Diagram illustrates crossing scheme used to obtain F3 populations (F3 pedigree). (*B*) Jittered dots depict % DNA methylation of individual plants at DMR1 determined by McrBC-qPCR. Plants denote F3 siblings–4-wk-old leaf tissue. The summary of the data is shown as horizontal line indicating the median. Gray boxes illustrate the data range. The × axis refers to *NRPE1* genotypes. *NRPE1/NRPE1 TAB2^sulf^, n* = 35; *NRPE1/nrpe1 TAB2^sulf^, n* = 94; *nrpe1/nrpe1 TAB2^sulf^, n* = 12. *P* value *nrpe1/nrpe1* versus *NRPE1/NRPE1* was calculated employing a Mann-Whitney–Wilcoxon test. (*C*) Jittered dots depict relative *TAB2* expression in individual plants (4-wk-old leaf tissue) normalized to the geometric mean of the expression of two reference genes (*SI Appendix*, Table S3). The summary of the data is shown as horizontal line indicating the median. Gray boxes illustrate the data range. F3 plants: *NRPE1/NRPE1 TAB2^sulf^, n* = 6; *NRPE1/nrpe1 TAB2^sulf^, n* = 4; *nrpe1/nrpe1 TAB2^sulf^, n* = 4. Controls: M82- (*S. lycopersicum cv. M82) NRPE1/NRPE1 TAB2^+^, n* = 2*; sulf-* (*S. lycopersicum cv. Lukullus) NRPE1/NRPE1 TAB2^sulf^, n* = 2*; nrpe1-nrpe1/nrpe1 TAB2,^+^ n* = 2. (*D*) Young leaves from 6-mo-old plants. White bar, 2 cm. (*E*) DMR1 sRNA size distribution in counts per million (CPM) in F3 plants and controls (4-wk-old leaf tissue). Genotypes are the same as in Fig. 2*C*. Jittered dots represent individual plants. The summary of the data is shown as horizontal line indicating the median. Errors bars represent the SD. Genotypes are the same as in Fig. 2*C*. (*A*–*E*) Yellow boxes refer to plants displaying *sulf* chlorosis, and green boxes refer to green plants.

To assess a possible role of an RdDM cofactor, we crossed *sulf* plants with a mutation in an RdDM component DNA-directed RNA NRPE1 (Fig. 2*A*) (19) and then selfed F1 plants that were epiheterozygous (*TAB2^sulf/+^*) (Fig. 2*A* and *SI Appendix*, Fig. S6*A*) for two generations. We were unable to obtain progeny with a maternal *nrpe1* mutation; however, the parent of origin does not affect *sulf* paramutagenicity (20), and the lack of reciprocal crosses would not compromise our interpretation.

The *nrpe1* mutant has a deletion of two codons in the *NRPE1* coding sequence, reduced 24-nt sRNAs, and hypomethylation at RdDM loci (19). In the F2 and F3 progeny, the DMR1 DNA methylation profiles were similar in all *NRPE1* genotypes (Fig. 2*B* and *SI Appendix*, Fig. S6*B*). In the F2, the mean level was close to 50% with a distribution from 0 to 100% (*SI Appendix*, Fig. S6*B*). In the F3, all of the plants had the *TAB2^sulf^* epiallele since DMR1 methylation was above 50% in all individuals regardless of their *NRPE1* genotype (Fig. 2*B*). On average, the F3 *nrpe1* plants had 20% lower DMR1 DNA methylation levels than their *NRPE1* F3 siblings (Fig. 2*B*), but this reduction did not suppress silencing of *TAB2* expression (Fig. 2*C*) or the *sulf* chlorosis (Fig. 2*D* and *SI Appendix*, Fig. S7). From these data, we conclude that reduced NRPE1 function has a minor effect on DMR1 DNA methylation but that PolV is not an essential cofactor of *sulf* silencing.

The 24-nt sRNAs constitute a hallmark of RdDM activity (9). Given the nonessential role of NRPE1 in maintaining *sulf*, we reassessed sRNAs at the *TAB2* locus employing sRNA sequencing. On average, 24-nt sRNAs accumulated more in *TAB2^sulf^* than in *TAB2^+^* at DMR1 (16) but at a low and variable level (*SI Appendix*, Fig. S8*A*). Gene body sRNAs showed a more consistent up-regulation in *sulf* plants (*SI Appendix*, Fig. S8*B*).

Whole-genome sRNA-sequencing data revealed that 13% of all sRNA loci were affected by NRPE1 (19), but in F3 *NRPE1* and F3 *nrpe1* plants, the low levels of DMR1 24-nt sRNA were not significantly different (Fig. 2*E*). Similarly, in *CMT3* and *cmt3* F2 plants of the crosses with *TAB2^sulf^,* the DMR1 sRNA of all 21- to 24-nt size classes was unaffected by genotype or *sulf* epigenotype (*SI Appendix*, Fig. S9 *A*–*C*). There was a reduction of gene body sRNAs in *cmt3* genotypes with *TAB2^+^* (*SI Appendix*, Fig. S9*D*), but this effect did not correlate with *sulf* chlorosis: DNA methylation was abundant in plants with *TAB2^+^* epigenotype (Fig. 1*G* and *SI Appendix*, Fig. S3). From these sRNA data, it is therefore unlikely that DMR1 or gene body sRNAs play a role in *sulf* maintenance.

### KYP and H3K9me2 Are Required to Maintain the *sulf* Paramutation.

In *Arabidopsis*, a self-reinforcing loop model involving H3K9me2, a repressive histone modification, can account for maintenance of CHG methylation by CMT3. In this model, KYP family proteins bind CHG DNA methylation and methylate histone H3K9 tails. In turn, *CMT3* binds H3K9me2 and increases the existing level of CHG DNA methylation (21, 22). Given the signature of CMT3-dependent DNA methylation at *TAB2*, the CMT3/KYP reinforcement model predicts higher levels of H3K9me2 in *sulf* than in WT tissues at DMR1.

**Fig. 3.**
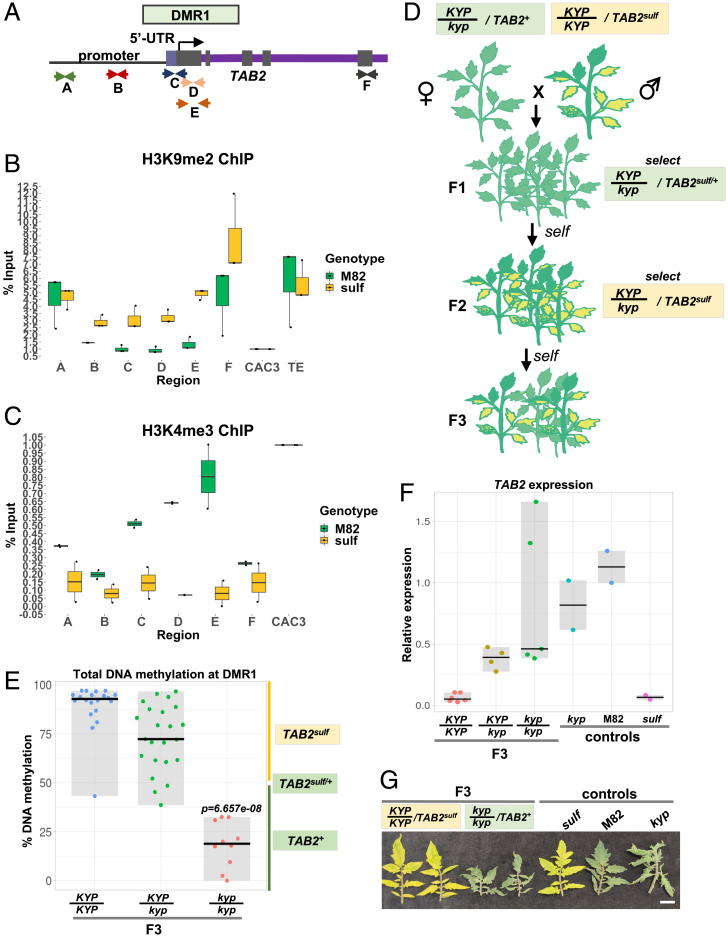
KYP maintains *sulf*. (*A*) Diagram represents the relative oligonucleotide position (arrows) spanning the *TAB2* locus used for ChIP-qPCR experiments in Fig. 3*B*. The different letters represent the different *TAB2* locus regions. (*B*) Box plot depicts H3K9me2 enrichment per % input normalized to *CAC3* reference locus determined by Chip-qPCR. Jittered dots represent different biological replicates. M82- *S. lycopersicum* cv. M82 *TAB2^+^* (green), *n* = 3; *sulf*- *S. lycopersicum cv. Lukullus* *TAB2^sulf^* (yellow), *n* = 3. The summary of the data is shown as horizontal line indicating the median of biological replicates. Error bars represent the SD. TE refers to T135 retrotransposon element. (*C*) Box plot depicts H3K4me3 enrichment per % input normalized to *CAC3* reference locus determined by Chip-qPCR. Jittered dots represent different biological replicates. M82- *S. lycopersicum* cv. M82 *TAB2^+^* (green), *n* = 2; *sulf*- *S. lycopersicum cv. Lukullus* *TAB2^sulf^* (yellow), *n* = 2. The summary of the data is shown as horizontal line indicating the median of biological replicates. Error bars represent the SD. (*D*) Diagram illustrates crossing scheme used to obtain F3 populations (F3 pedigree). (*E*) Jittered dots depict % DNA methylation at DMR1 individual plants determined by McrBC-qPCR. Plants denote F3 siblings. The summary of the data is shown as horizontal line indicating the median. Gray boxes illustrate the data range. The × axis refers to *KYP* genotypes. F3 plants: *KYP/KYP TAB2^sulf^, n* = 20; *KYP/kyp TAB2^sulf^, n* = 23; *kyp/kyp TAB2^+^, n* = 10. *P* value *kyp/kyp* versus *CMT3/CMT3* was calculated employing a Mann-Whitney–Wilcoxon test. (*F*) Jittered dots depict relative *TAB2* expression in individual plants normalized using the geometric mean of the expression values for two reference genes (*SI Appendix*, Table S3). The summary of the data is shown as horizontal line indicating the median. Gray boxes illustrate the data range. F3 plants: *KYP/KYP TAB2^sulf^, n* = 6; F3 plants: *KYP/kyp TAB2^sulf^, n* = 4; *kyp/kyp TAB2^+^, n* = 5. Controls: M82- (*S. lycopersicum cv. M82) KYP/KYP TAB2^+^, n* = 2*; sulf-* (*S. lycopersicum cv. Lukullus) KYP/KYP TAB2^sulf^, n* = 2*; kyp- kyp/kyp TAB2^+^, n* = 2. (*G*) Young leaves excised from 6-mo-old plants. White bar, 2 cm. (*A*–*G*) Yellow boxes refer to plants displaying *sulf* chlorosis, and green boxes refer to green plants. UTR, untranslated region.

To test this prediction, we carried out H3K9me2 chromatin immunoprecipitation (ChIP) followed by quantitative real-time PCR (ChIP-qPCR) with oligonucleotide pairs A–F spanning different regions of the *TAB2* locus (Fig. 3*A*) in M82 and *sulf* plants bearing *TAB2^+^* and *TAB2^sulf^* epigenotypes, respectively. We normalized the input using an unrelated reference locus. This approach allowed us to determine the relative H3K9me2 abundance but not the proportion of modified nucleosomes. Consistent with our prediction, the H3K9me2 enrichment was higher in *TAB2^sulf^* than in *TAB2^+^* at the DMR1 overlapping regions C, D, and E, including the *TAB2* TSS, and also at the promoter region B (Fig. 3*B*). In contrast, we detected no or smaller H3K9me2 differences between *TAB2^+^* and *TAB2^sulf^* at regions outside DMR1, including A and F and an unrelated TE (Fig. 3*B*). From these results, we conclude that the link between CMT3 and *sulf* is associated with increased levels of H3K9me2 at DMR1.

In M82 leaves bearing *TAB2^+^*, the H3K9me2 levels were lower close to the TSS than in more distal regions (Fig. 3*B*). Such low levels are exceptional in pericentromeric heterochromatin (where TAB2 resides) (18), and it is likely that these low H3K9me2 levels enable transcription of *TAB2*. Consistent with this interpretation, there were high levels of the active transcription mark H3K4me3 close to *TAB2* TSS (Fig. 3*C*) detected by ChIP-qPCR in M82 plants at regions C, D, and E. The association between histone modifications with *sulf* silencing is reinforced by reduced levels of the H3K4me3 mark at these sites at *TAB2^sulf^* (Fig. 3*C*).

To further test the involvement of H3K9me2, we crossed *sulf* with a mutant bearing CRISPR-Cas9–mediated deletion at the gene encoding for the *Solanum lycopersicum* KYP (18) (Fig. 3*D*). The crossing strategy was parallel to that used for *cmt3*, and in the F2 and F3 progeny of the F1 *KYP/kyp* epiheterozygous *TAB2^sulf/+^* (*SI Appendix*, Fig. S10*A*), the *kyp/kyp* plants all had less than 50% DMR1 DNA methylation (*SI Appendix*, Fig. S10*B* and Fig. 3*E*). Accordingly, *TAB2* expression in F3 *kyp* plants was higher than in the *KYP* siblings (Fig. 3*F*), and none displayed symptoms of chlorosis (Fig. 3*G* and *SI Appendix*, Fig. S11). Only progeny that carried an active *KYP* allele displayed *TAB2^sulf^* epigenotype, *sulf* phenotypes, and low *TAB2* expression (Fig. 3 *E* and *F*), as observed in the original *TAB2^sulf^* parent. The *sulf* paramutation in tomato, therefore, is associated with high levels of CHG DNA methylation and H3K9me2 at DMR1 that would be expected to influence chromatin organization.

### Chromatin Architecture Changes in *sulf*.

To test this hypothesis, we carried out chromatin conformation capture (Hi-C) analysis in *TAB2^sulf^* and *TAB2^+^*. Despite having distinct leaf colors, M82 and *sulf* plants showed highly similar Hi-C maps, suggesting that there were no intensive genome rearrangements or changes in chromatin contacts in *sulf* plants bearing *TAB2^sulf^* epialleles (*SI Appendix*, Fig. S12 *A* and *B*). Principal component analysis (PCA) of the contact matrix of chromosome 2 revealed A/B compartments that correlated to the delineation of euchromatin/heterochromatin across this chromosome (*SI Appendix*, Fig. S13 *A* and *B*). This A/B compartment partition was broadly similar in the two epigenotypes, and in all the samples, the genomic region containing *TAB2* belonged to the heterochromatic B compartment (*SI Appendix*, Fig. S13*C*). However, there was a change in the PCA eigenvalue of this genomic region in all the *sulf* replicates, indicating a change in chromatin interaction between this region and the A/B compartments. By examining the interaction strength between this *TAB2*-containing region and the A compartment, we found that it was significantly weaker in the *TAB2^sulf^* plants than in the *TAB2^+^* plants (*SI Appendix*, Fig. S13*D*), indicating a general shift away from euchromatic regions.

## Discussion

We confirm here that *sulf* paramutation is associated with DMR1 that overlaps the TSS of *TAB2* (16) (Fig. 1*G* and *SI Appendix*, Fig. S3), and we have shown additionally the involvement of CMT3/KYP (Figs. 1 and 3) and an associated change in chromatin organization (*SI Appendix*, Fig. S13). CMT3/KYP have not been previously associated with endogenous gene paramutation, although in *Arabidopsis*, a *LUC* paramutation transgene relies on multiple proteins including MET1 and CMT3/KYP (23). The involvement of CMT3 in maize is difficult to test because loss of function in the *ZMET2* and *ZMET5* homologs is not compatible with plant viability (24). However, the CHG subcontext methylation of B’, a highly paramutagenic maize allele at the *b1* locus, has the CMT3 signatures (*SI Appendix*, Fig. S14). This pattern is consistent with *ZMET2* and *ZMET5* playing a role in the maintenance of *b1* paramutation in maize. In addition, there are high H3K9me2 levels correlated with *b1* and *p1* paramutagenic alleles (25, 26). As with the subcontext data, this feature is consistent with a CMT3/KYP self-reinforcing loop in paramutation in maize as well as tomato.

The dynamics of *sulf* paramutation vary depending on the *sulf* epiallele used as parent. Some *sulf* epialleles transfer the repressive state in the F1, but with the *sulf* epialleles used here, the transfer likely occurs in the F2 (17). The *sulf* epiallele was transmitted and maintained in the *CMT3*/*CMT3* homozygous F1 or *CMT3*/*cmt3* heterozygous F1 and then progressively transferred between *TAB2* alleles in the *CMT3*/*CMT3* or *CMT3*/*cmt3* F2 but not in the *cmt3*/*cmt3* homozygous F2. While we cannot rule out that F2 plants with the *sulf* phenotype had inherited two silent alleles of *TAB2*, it is known that *TAB2^sulf^* homozygotes die at the seedling stage (5); therefore, a more likely scenario is that they germinated as *TAB2^sulf/+^* and then progressed to *TAB2^sulf^* through paramutation shortly after germination.

**Fig. 4.**
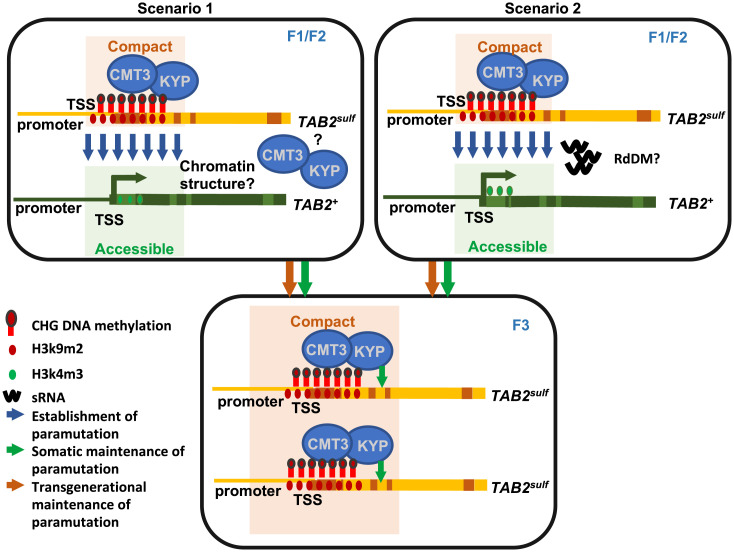
Model to explain the establishment (F1 or F2) and maintenance of the *sulf* paramutation in tomato. *Scenarios 1* and *2* represent alternative hypotheses to explain the establishment of the *sulf* paramutation, which are not mutually exclusive. In *Scenario 1*, the establishment of paramutation requires a CMT3/KYP–dependent compact chromatin conformation, which in turn facilitates the exchange of epigenetic marks. In *Scenario 2*, the initial round of DNA methylation at *TAB2* is initiated by RdDM and sRNAs. We show that CMT3/KYP is required either to complete silencing or/and maintain *TAB2^sulf^* in subsequent cell divisions and across generations. Yellow/orange boxes represent the silenced paramutagenic epiallele *TAB2^sulf^,* which exists in a compact chromatin conformation. Green boxes represent the active paramutable *TAB2^+^* epiallele in which chromatin exists in a less compact structure and therefore remains more accessible to the transcription machinery.

One scenario is that CMT3/KYP is required to make the *TAB2^+^* chromatin sensitive to receive the paramutation silencing signal from a silenced allele (Fig. 4, *Scenario 1*). Alternatively, the paramutagenic allele (*TAB2^sulf^*) silences the paramutable allele (*TAB2^+^*) in the *cmt3* homozygote, as it would do so in the *CMT3* genotype; however, the silencing is not maintained, as *CMT3* is needed to either complete the *sulf* silencing process or to allow the repressed state to persist (Fig. 4, *Scenario 2*). In the former scenario, the role of *CMT3* would be in establishment, and in the latter, it would be in maintenance of paramutation (Fig. 4).

The involvement of KYP and the changes to the chromatin modifications and organization of *TAB2* in *sulf* are consistent with all of these hypotheses. However, the finding that CMT3 is absolutely required to maintain *sulf* epigenetic memory (*SI Appendix*, Figs. S4 and S5) implies a role of CMT3/KYP and the associated change in chromatin architecture in maintenance. This finding contrasts with demonstration that paramutated states persist through backcrosses with maize RdDM mutants (10). The various *CMT*3 hypotheses are not, however, mutually incompatible because *TAB2* methylation could be lost directly in *cmt3* gametes in F2 plants or during F3 early development in *cmt3/cmt3* plants.

The accepted paradigm for establishment of paramutation invokes sRNAs in the communication between interacting alleles. In our *sulf* system, however, there is a very low level of (24-nt) sRNA accumulation at *TAB2^sulf^* (*SI Appendix*, Fig. S8) (16), and loss or reduced function of PolV does not lead to loss of *sulf* silencing (Fig. 2). These findings are not easy to reconcile with a simple role of RdDM in the maintenance of *sulf* paramutation as previously described (16), although we cannot rule it out conclusively in terms of establishment (Fig. 4). It could also be that there is residual PolV function in the mutant line and that *sulf* sRNA is abundant at specific developmental stages when communication between alleles takes place. Although unlikely, we cannot also exclude that in *nrpe*1 pollen (used here to introgress *nrpe1* in the *sulf* background) sRNA populations are less affected than maternal sRNAs (27), thereby decreasing the effect of NPRE1 loss in the *sulf* phenotype.

Even in maize, however, the 24-nt sRNA role in paramutation also remains unclear. For instance, RMR1 and RMR7 are not required for paramutation establishment at Pl1-Rhoades despite affecting 24-nt sRNA accumulation (13, 14). In addition, paramutation is affected by genetic backgrounds with normal 24-nt sRNA levels (15). Furthermore, sRNA accumulation is not sufficient to direct paramutation at *b1*, indicating that other factors must be involved (28).

In summary, our results indicate that models of paramutation should accommodate the involvement of CMT3/KYP and changes in chromatin structure in at least the maintenance/memory phase if not in establishment (Fig. 4). These models should be open to the possibility that there could be locus-specific and/or species-specific mechanisms of paramutation and that there could be communication between alleles by mechanisms other than through sRNA and RdDM. Further understanding of chromatin organization and the 4D genome will also be necessary to understand how paramutation, uniquely among epigenetic phenomena, enables communication between alleles.

## Materials and Methods

### Plant Growth.

Plants were germinated and grown in *F2* compost and transferred to *John Innes 2* compost 3 wk after germination. Plants were grown in 16-h light (22 °C) and 8-h dark (18 °C) cycles with 70% humidity and light intensity 300 µmol l× m^−2^ × s^−1^ photosynthetically active radiation (PAR). Extended plant methods and genotyping details are provided in the *SI Appendix*.

### McrBC Digestion.

Genomic DNA was isolated from 100 mg leaf tissue using DNeasy Plant Mini Kit (Qiagen) according to the manufacturer’s instructions. The DNA samples used for McrBC digestion were the same as the samples used for genotyping. McrBC digestion was performed as described in (29). Details about qPCR are provided in the *SI Appendix*.

### Expression Analysis.

Total RNA isolation was performed with the Direct-zol RNA Miniprep (Zymo Research) and TRIzol reagent (Invitrogen) according to the manufacturer’s instructions. For reverse-transcription qPCR analyses, 1 μg of total RNA was reverse transcribed using RevertAid First Strand cDNA Synthesis Kit (Thermo Fisher Scientific) according to the manufacturer’s instructions with random hexamer primers. Details about qPCR are provided in the *SI Appendix*.

### ChIP.

Chromatin extraction was carried out as described in (18) using 1 g of leaf tissue of 4-wk-old plants. Chromatin was fragmented between 200 and 600 bp in a Covaris E220 evolution (duty cycle: 20%, peak intensity: 140, cycles of burst: 200, time: 3 min). Immunoprecipitation was carried out as described in (18) with Anti-Histone H3 (di methyl K9) antibody–ChIP Grade (Abcam, ab1220) and Anti-Histone H3 (tri methyl K4) antibody–ChIP Grade (Abcam, ab8580). The material was reverse cross-linked by adding NaCl to a final concentration of 200 mM and incubating at 65 °C overnight. This was followed by a 30-min treatment with 1 μL RNase A (Thermo Fisher Scientific) at 37 °C and a 90-min treatment with 1.5 µL Proteinase K (Thermo Fisher Scientific) at 65 °C. DNA was purified using the MinElute Kit (Qiagen) according to manufacturer’s instructions and eluted in 35 µL. Details about qPCR are provided in the *SI Appendix*.

## Supplementary Material

Supplementary File

## Data Availability

Sequencing data have been deposited in Array Express (accession Nos. E-MTAB-10556, E-MTAB-10557, E-MTAB-10565, E-MTAB-10568, and E-MTAB-10574). All other study data are included in the article and/or *SI Appendix*. Previously published data were used for this work (DOI: 10.1093/jxb/erw096 BioProject SRP066362).
